# Higher Visual Function Deficits in Children With Cerebral Visual Impairment and Good Visual Acuity

**DOI:** 10.3389/fnhum.2021.711873

**Published:** 2021-11-16

**Authors:** Arvind Chandna, Saeideh Ghahghaei, Susan Foster, Ram Kumar

**Affiliations:** ^1^The Smith-Kettlewell Eye Research Institute, San Francisco, CA, United States; ^2^Alder Hey Children’s Hospital, Liverpool, United Kingdom

**Keywords:** higher visual function deficits, screening, questionnaire, good visual acuity, children, low vision, cerebral visual impairment (CVI), structured question inventory

## Abstract

In clinical practice Cerebral Visual Impairment (CVI) is typically diagnosed by observation of abnormal visually guided behaviors which indicate higher visual function deficits (HVFDs) suggesting abnormal brain development or brain damage in a child with a suitable clinical history. HVFDs can occur even in the presence of good visual acuity and may remain undiagnosed *because* the good visual acuity does not prompt further investigation. This leads to a lack of understanding of the child’s visual perceptual difficulties. In a prospective study, we determined the spectrum of HVFDs in a group of children with history suggestive of brain damage or disruption of brain development and an independent diagnosis of CVI in comparison with typically developing children with a structured 51 question inventory, the Higher Visual Function Question Inventory (HVFQI-51) adapted from the Cerebral Vision Impairment Inventory, CVI-I. Here, we show that the HVFQI-51 can detect a range of HVFDs in children with CVI with good visual acuity and clearly distinguishes these children from typically developing children. HVFDs in our study group could mostly be attributed to dorsal stream visual processing dysfunction though the spectrum varied between children. We report on the inclusion of the “not applicable” response option in analysis providing a picture of HVFDs more in tune with the overall disability of each child. We also propose a subset of 11 questions (Top-11) which discriminate between children with CVI vs. behaviors seen in typical children: this provides both a *potential* screening tool for initial assessment of HVFDs and a measure of CVI-related impairment, and needs further validation in a secondary independent sample.

## Introduction

Cerebral Visual Impairment (CVI) is a heterogenous disorder of brain-based visual impairment resulting from brain injury or disruption of development of retrochiasmatic visual pathways and vision processing regions of brain, commonly occurring during gestation at or around birth. In clinical practice, CVI is typically diagnosed in a child with a suitable clinical history by observation of abnormal visually guided behaviors (i.e., behaviors that rely on normal visual function) that suggest abnormal brain development or brain damage. These behaviors can stem from higher visual function deficits (HVFDs) of visual processing with consequent perceptual deficits, even in the presence of normal or near-normal visual acuity (Dutton and Jacobson, 2001; Fazzi et al., 2007, 2009; Saidkasimova et al., 2007; Boot et al., 2010; van Genderen et al., 2012; Philip and Dutton, 2014). However, HVFDs, in presence of good visual acuity, often remain undiagnosed because good visual acuity precludes further investigation leading to a lack of understanding of the child’s visual perceptual difficulties. The reasons are mainly historical. CVI, previously termed cortical blindness (Marquis, 1934) and later Cortical Visual Impairment (Whiting et al., 1985) was previously diagnosed based on severity of visual acuity loss which limited the understanding of the condition (Hoyt and Fredrick, 1998; Frebel, 2006; Colenbrander, 2010). It is now clear that manifestations of this condition involve more than the occipital cortex and CVI is associated with a spectrum of agnosias indicating presence of HVFDs, oculomotor abnormalities and secondary changes in the optic nerve have been documented (Jacobson et al., 1998; Jacobson and Dutton, 2000; Jan et al., 2001; Salati et al., 2002; Dutton et al., 2004). In the light of these findings, CVI, by consensus, is now termed *Cerebral* Visual Impairment, “a verifiable visual dysfunction which cannot be attributed to disorders of the anterior visual pathways or any potentially co-occurring ocular impairment” (Sakki et al., 2018) with HVFDs synonymous with visual perceptual difficulties (Vancleef et al., 2020). Using visual acuity criterion alone is likely to miss a large proportion of children with HVFDs and a diagnosis of CVI should be based on the combined presence of multiple factors with reduced visual acuity being a contributory but not the defining criteria. Tsirka et al. (2020) using the Insight question inventory for detecting HVFDs in children aged 5–16 years applied the following eligibility criteria for CVI: (i) a confirmed diagnosis of CVI based on a known medical reason for brain injury or dysfunction, (ii) no signs of ocular pathology other than mild optic atrophy (defined by indirect ophthalmoscopy), and (iii) binocular visual acuity of at least LogMAR 1.0 (Snellen 6/60); relegating visual acuity loss to one of the criteria. The diagnosis is based on an assessment of clinical history of predisposing factors, parental reports of visual behaviors suggestive of HVFDs, an ocular examination to exclude a purely ocular cause of the visual impairment (though CVI may co-exist with an ocular condition especially when associated with prematurity such as retinopathy of prematurity, optic nerve hypoplasia; for a review see Fazzi et al., 2007], input from a neurological examination and, when possible, supplemented by other investigations such as neuroimaging. Normal visual acuity and absence or presence of neuroimaging findings no longer excludes a diagnosis of CVI (Stiers et al., 2002; Bassan et al., 2007; Saidkasimova et al., 2007; Fazzi et al., 2009; Ortibus et al., 2009, 2012; van Genderen et al., 2012).

Visually guided behaviors and cognitive processes or Higher Visual Functions (HVFs) are best explained through a functional model of two cerebral networks comprising the dorsal stream connecting occipital V5 (area MT), V3A areas and parietal lobes; and the ventral stream connecting the occipital and inferotemporal (area IT) cortical area (Felleman and Van Essen, 1991; Young, 1992). Visual functions such as motion perception, dealing with complex visual scenes, navigation through three dimensional space and visually guided movements are assigned to the dorsal stream—the “where” or “action” pathway of HVFs; while color, shape, object, word and face recognition are assigned to the ventral stream—the “what” stream of HVFs (Mishkin et al., 1983; Goodale and Milner, 1992; Milner and Goodale, 2008; Goodale, 2013). In early life, the functional morphology of the brain representing the dorsal stream is thought more vulnerable (Braddick et al., 2003) resulting in a preponderance of dorsal stream visual function deficits in CVI (Macintyre-Beon et al., 2013). There is, however, considerable overlap between the two putative streams in the execution of most visual functions such as identification of objects and visually guided motion to reach and grasp (Milner, 2017).

The detection of HVFDs is difficult as young children with CVI cannot self-report and, older children are usually agnostic of their HVFDs as they have not lost an ability—they never developed the function (*they know not what they know not*). In addition, the presence of good visual acuity which often precludes further investigations (Sakki et al., 2018), the lack of readily available tools (Gorrie et al., 2019) or the knowledge and understanding of manifestations of CVI amongst clinicians and teachers (Fielder et al., 1993; Youngson-Reilly et al., 1994; McDowell, 2020) adds to the challenges of identifying HVFDs. However, diagnosing HVFDs is essential since they can cause significant visual disability in everyday activities and education especially, while visual acuity remains largely intact (Mercuri et al., 1998; Dutton and Jacobson, 2001; Fazzi et al., 2007; Saidkasimova et al., 2007; van Genderen et al., 2012).

We chose a structured history taking tool (The CVI Inventory, CVI-I) that was designed to be used by clinicians to record parental observations in order to assess and document HVFDs which might otherwise go unnoticed in children with CVI (Dutton et al., 2010b). Furthermore, based on the responses, the CVI-I provides guidance to implement (re)habilitation strategies for HVFDs. This inventory (51 questions) and its previous version of 52 questions (the Insight Inventory) has been validated in children with CVI with moderate to severe visual acuity loss (Houliston et al., 1999; Dutton, 2011; Macintyre-Beon et al., 2012; Philip et al., 2016; Sakki et al., 2020; Tsirka et al., 2020). Though the diagnosis of CVI was not established by independent criteria in most of the studies except by Macintyre-Beon et al. (2012) (see section “Discussion”), the results were encouraging and further studies were recommended. However, prospective studies on children with an independent diagnosis of CVI and normal visual acuity are lacking at present in the published literature with only one retrospective study (van Genderen et al., 2012) reporting on children with good visual acuity and CVI and with suspected CVI using an abbreviated question inventory, adapted from the question inventory of Houliston et al. (1999). Their results showed CVI remains a clinical diagnosis and their question inventory should only be used to identify “symptoms” associated with CVI. Several other questionnaires and modifications of the questionnaires have been developed and utilized to detect HVFDs in children with CVI (Ortibus et al., 2011; van Genderen et al., 2012; Geldof et al., 2015; Salavati et al., 2017; Ben Itzhak et al., 2019; Gorrie et al., 2019; Fazzi and Micheletti, 2020, and for a recent review see McConnell et al., 2021).

The primary goal of this prospective study was to characterize the range of HVFDs in children with good visual acuity in the presence of an independent clinical diagnosis of CVI compared to a typical group of children. The CVI population with good visual acuity is most at-risk of HVFDs not being identified. We adapted the CVI Inventory (CVI-I) with its 51 questions (Bax, 2010) with permission from the original author Dr. Gordon Dutton, making minor changes and have used the name Higher Visual Function Question Inventory (HVFQI-51) to ensure clarity of purpose: (i) to determine the spectrum of HVFDs in a group of children with a history suggestive of brain damage or disruption of brain development and (ii) not use the HVFQI to diagnose “CVI” in these children; instead we used it in this group of children who were already diagnosed with CVI (van Genderen et al., 2012). We also investigated the most reliable questions in the HVFQI-51 and outline them in a new shortened inventory that may potentially serve as a short screener or as a measure of CVI-related impairment. Further, we sought to determine how best to score the inventory accounting for the responses to the “*Not Applicable”* option (NA), which have not been dealt with in previous publications using this inventory. In our experience, NA is a useful response option since a number of the observed behavior items may not be developmentally appropriate for the age of the child or for other reasons such as comorbid impairments. We allowed the parents to use the NA option and comment on their analysis and utility within the questionnaire.

## Materials and Methods

### Participants

Study participants were recruited through the patient population of the Alder Hey Children’s Hospital, Liverpool, United Kingdom (AH). This study received ethical approval NHS Research Heath Authority IRAS ID:193481; REC Reference:16/EE/0062 and abided by the tenets of the Declaration of Helsinki. Informed written consent was obtained from parents and assent from children where appropriate. Children with CVI were recruited after a diagnosis of CVI was established (see section below on CVI diagnosis) from the eye and neurology departments. Typically developing children were recruited from verbal requests largely through parents of children undergoing routine screening in the community and some from colleagues and friends. All participants (parents and assenting children) were naive to the purpose of the HVFQI and the design of the study.

Participants included 33 children with CVI with good binocular visual acuity and 111 typically developing children. The mean age (±SD) of participants was 7.0 years (±2.8) for the CVI group and 8.7 years (±2.8) for the typically developing group. The average crowded Lea Symbol binocular visual acuity was 0.14 ± 0.12 LogMAR for the CVI group with only 3 children with acuity worse than 0.2 LogMAR (but better 0.4 LogMAR) indicating good visual acuity in the presence of a diagnosis of CVI; and 0.14 ± 0.16 LogMAR for the typically developing group (see Table 1). Visual acuity in the typically developing group is lower than expected normal acuity reflecting the effect of using crowded acuity charts (Atkinson et al., 1986; Huurneman et al., 2012a,b; Anstice and Thompson, 2014).

**TABLE 1:** Clinical characteristics of children with CVI.

ID	Gender	Age	Term/weeks	VAOU	Amblyopia	Ref Error	Strab	ONH OD	ONH OS
1	F	5.75	35	0.00	None	0	0	Mild pallor	Normal
2	F	6.80	Term	0.10	OS	1	1	Normal	Normal
3	M	11.17	Term	0.00	OD	1	1	Normal	Normal
4	M	5.91	27	0.20	OU	1	0	Normal	Normal
5	F	12.51	Term	0.00	None	0	0	Normal	Normal
6	F	14.02	34	0.20	OU; OS > OD	0	1	Normal	Normal
7	M	6.93	34	0.10	OD	1	0	Normal	Normal
8	F	8.51	Term	0.20	OU	1	1	Normal	Normal
9	F	5.78	34	0.20	OU; OD > OS	0	1	Normal	Normal
10	F	5.72	Term	0.20	OU; OS > OD	1	1	Pallor	Pallor
11	F	10.12	27	0.10	OU	1	1	Normal	Normal
12	F	10.18	34	0.10	OU; OD > OS	1	1	Normal	Normal
13	M	11.36	Term	0.20	OU; OD > OS	1	0	Mild pallor	Mild pallor
14	M	8.50	Term	0.10	None	1	1	Temp pallor	Temp pallor
15	F	11.57	33	0.20	OU	1	0	Normal	Normal
16	F	10.66	Term	0.10	OU; OS > OD	1	0	Hypoplasia	Hypoplasia
17	M	9.72	Term	0.30	OU; OS > OD	1	1	Mild temp pallor	Slight temp pallor
18	F	5.67	Term	0.20	OU; OD > OS	1	1	Mild pallor	Normal
19	F	12.14	29	−0.10	None	0	1	Normal	Normal
20	M	4.52	Term	0.40	OU	1	1	Normal	Normal
21	M	10.82	Term	0.00	None	0	1	Normal	Normal
22	F	7.38	Term	0.00	None	1	0	Normal	Normal
23	M	4.44	38	0.10	None	0	1	Normal	Normal
24	F	11.56	Term	0.10	OS	1	1	Pallor	Pallor
25	M	9.09	Term	0.20	OU; OS > OD	0	1	Normal	Hypoplasia
26	F	8.32	Term	0.00	None	0	1	Normal	Normal
27	M	14.53	33	0.00	OS	1	1	Normal	Normal
28	F	8.05	30	0.20	OU	0	1	Normal	Normal
29	F	4.41	35	0.10	None	0	0	Poor views	Poor views
30	M	5.35	Term	0.20	OU	0	1	Normal	Normal
31	F	8.81	33	0.10	None	0	1	Mild temp pallor	Slight temp pallor
32	M	9.27	Term	0.20	OU	0	1	Pallor	Pallor
33	M	9.42	30	0.30	OU	0	1	Normal	Normal

*Gender, age at the time of the test, Binocular Visual Acuity (VAOU; Lea Symbol Test) presence/absence of amblyopia (OU, Bilateral; OD, Right Eye; OS, Left Eye; OS > OD and similar indicates amblyopia in left eye worse than right eye), refractive error (Ref Error) and strabismus (Strab) is reported. (ONH, Optic Nerve Head; temp, temporal) ONH status is reported for each eye separately. We also report neurological diagnoses and MRI findings (see Table 2). ONH, Optic Nerve Head.*

### Diagnosis of Cerebral Visual Impairment

Diagnosis of CVI was based on an integrated assessment of gestational, birth and developmental history; detailed eye, oculomotor and sensory status examination including cycloplegic refraction (AC, SF; optometrist); detailed neurologic examination and review of neuroimaging for clinico-radiological diagnosis (RK); symptom correlation (AC, RK) and, MRI in almost all (31 out of 33) children (see Tables 2, 3). Typically developing children were declared normal based on detailed history which included detailed birth and developmental history and an eye examination which included normal distance visual acuity, normal ocular and sensorimotor status and, non-dilated retinal examination. Oculomotor status was assessed with cover tests (Cover-Uncover and Alternate-Cover test); assessment of extraocular movements; sensory status was determined with age-appropriate tests for stereopsis (Frisby; TNO; Lang Stereotest) and fusion (Bagolini Striated lenses and Worth 4-dot test) by experienced clinicians (AC and SF).

**TABLE 2:** Neurological summary diagnoses and Brain-MRI scan results of children with CVI.

ID	Primary neurological diagnosis	Brain MRI findings
1	16p13.11 deletion syndrome	Normal
2	Cerebral palsy, GMFCS Level 2, asymmetric spastic diplegia	PVL
3	Global Developmental Delay, ASD, mild neurodevelopmental deficits	normal
4	Global developmental Delay, ASD	PVL
5	ASD, ADHD	Not available
6	Neonatal meningitis	PVL
7	Learning difficulties (moderate), ASD	Normal
8	Cerebral palsy, GMFCS Level 3, spastic diplegia	Not available
9	Cerebral palsy, GMFCS Level 3, spastic diplegia	PVL (performed elsewhere)
10	Congenital Achiasma Syndrome (abnormal VEP)	Normal
11	Cerebral palsy, GMFCS Level 1, left hemiplegia	Right frontal porencephalic cyst
12	Severe IUGR, dyspraxia, feeding difficulties, joint hypermobility	PVL
13	Cerebral palsy, GMFCS Level 4, asymmetric spastic quadriplegia, right side more involved	Left frontoparietal porencephalic cyst, hydrocephalus
14	Cerebral palsy, GMFCS Level 2, mild neurodevelopmental deficits	Right frontoparietal porencephalic cyst, white matter volume loss
15	Global Developmental Delay, moderate learning difficulties	PVL
16	Cerebral palsy, GMFCS Level 1, right hemiplegia, mild learning difficulties, newborn HIE Grade 3	Bilateral occipital gliosis
17	Newborn symptomatic hypoglycemia, normal gross neurology	Bilateral occipital gliosis
18	Neurodevelopmental and congenital cardiac malformation syndrome, severe learning difficulties	PVL
19	Social communication difficulties, dyspraxia	Normal
20	Cerebral palsy, GMFCS Level 1, right hemiplegia	Left fronto-parietal porencephalic cyst
21	Neonatal hemorrhagic stroke, normal gross neurology	Right Occipital Gliosis
22	IUGR, ASD	Normal
23	ASD	Normal
24	Cerebral palsy, GMFCS Level 2, right hemiplegia	Left temporo-parietal porencephalic cyst
25	ASD	Bilateral Dilated Ventricles
26	Meningitis, hydrocephalus	Hydrocephalus
27	Normal gross neurology	PVL
28	Cerebral palsy, GMFCS Level 3, spastic diplegia	PVL
29	Fine motor impairment, Behavioral disorder	PVL
30	Cerebral palsy, GMFCS Level 2, spastic diplegia, mild learning difficulties, neonatal meningitis	Bilateral occipital and parietal gliosis
31	Neonatal arterial ischemic stroke	Left parietal and temporal multicystic encephalomalacia
32	Cerebral palsy, GMFCS Level 2, spastic diplegia, traumatic perinatal intracerebral hemorrhage	Right temporal and parietal multicystic encephalomalacia
33	Neonatal Meningitis	Left occipital gliosis and atypical PVL

*MRI was not available in child #5 and #8. GMFCS, Gross Mototr Function Classification System; ASD, Autistic Spectrum Disorder; ADHD, Attention Deficit Hyperactivity Disorder; IUGR, Intrauterine Growth Restriction; HIE, Hypoxic Ischemic Encephalopathy; PVL, Periventricular Leukomalacia; VEP, Flash and Pattern Reversal Visual Evoked Potential.*

**TABLE 3:** Endorsement criteria for the dichotomies.

Dichotomy name (Likert scale score)	Not endorsed: Score of 0	Endorsed: Score of 1
“Rarely”, (1)	Never	Rarely, Sometimes, Often, and Always
“Sometimes”, (2)	Never and Rarely	Sometimes, Often, and Always
“Often”, (3)	Never, Rarely, and Sometimes	Often and Always
“Always”, (4)	Never, Rarely, Sometimes, and Often	Always

*Dichotomies were named based on the cutoff response level. The 5-point Likert scale score corresponding to the name of the dichotomy name is also provided (range 0 = “Never” to 4 = “Always”).*

### Question Inventory

The HVFQI comprised 51 questions (HVFQI-51) organized into clusters of questions that seek behavioral evidence of impairment of visual cognition, including putative dorsal and ventral stream dysfunction. The inventory was adapted from the CVI-I (Dutton et al., 2010b; Macintyre-Beon et al., 2012) and following modifications were made. We added instructions on completing the question inventory, a brief explanation of the purpose of the study (but not the purpose of the HVFQI) in accordance with our ethical approval. We also replaced the misprint in question 42 “Do quiet places/open countryside cause difficult behavior?” with “Do quiet places/open countryside result in better behavior?” (changed after personal communication with the author of the original questionnaire Dr. Dutton). The name of the question inventory was changed to HVFQI-51 to reflect the purpose of the question inventory—to document HVFDs and not use it to diagnose CVI.

The research project was explained to the parent or caregiver by the responsible clinician (SF or AC) in a standard format explaining the purpose of the research project and process of completing the QI. Queries were addressed without giving leading explanations or answers. The questions were answered in one sitting. Parents chose a response from a standard 5-point Likert scale (e.g., Never, Rarely, Sometimes, Often, Always). An additional “Not Applicable” (NA) option for each question was chosen only if a particular question could not be answered; for example, child was too young or a physical disability precluded applicability of that particular visual behavior.

### Analysis and Exploratory Analysis

Analyses were performed using Python (NumPy and SciPy libraries).^1^

#### Analysis of the Responses, Accounting for the Not Applicable Response

To determine the ability of the HVFQI-51 to distinguish between the two groups (children with CVI and typical children), values of 0, 1, 2, 3, or 4 were assigned to Never, Rarely, Sometimes, Often and Always, respectively. Therefore, higher scores reflect more impairment. A total score was calculated on these *applicable* responses, where answered, for each child; the *average* score for each of the 51 questions and for each group. For example, for a given child, if the number of applicable answers was 48, then the total score would be the average of the values assigned to these 48 questions.

#### Analysis for the Most Discriminatory Questions

We also wished to determine whether a particular response on the five-level ordinal-response Likert scale when compared against the other responses would endorse a subset of “most discriminatory” questions. The purpose of this analysis was to determine whether a set of fewer questions would lead to a *potential* screening tool, or a potential tool for measuring CVI-related impairment, discriminating from the normal range of behaviors seen in typical children.

For this analysis we employed a series of dichotomy analyses where we split the five-level ordinal responses into binary groupings (Table 3; column 1, 2, and 3). It is typical in analysis of a potential clinical tool to reduce the 5-point Likert scores to a dichotomy based on a fixed level for all questions: a response of Yes indicating ‘endorsed’ and No indicating ‘not endorsed’ (Houliston et al., 1999, see Dutton’s Top-5 in Dutton et al., 2010a), or the level that gives the best performance for a given question. First, the response “0” (not endorsed) was assigned to questions with the Never response and “1” (endorsed) was assigned to questions answered as any one of the four remaining four responses (Rarely, Sometimes, Often or Always); this was called the dichotomy of “Rarely.” Next, “0” was assigned to questions answered as Never or Rarely and “1” assigned to questions answered as one of the remaining 3 responses (Sometimes or Often or Always); this was called the dichotomy of “Sometimes”; and so on for a total of 4 cut-off points creating four dichotomies. Each dichotomy was then analyzed (Table 3).

## Results

### Not Applicable Responses

The frequency of NA responses was higher in the CVI group compared to the typical group: median number of 1 (75% quartile at 3 NAs) for the CVI group compared to 0 (75% quartile at 0 NAs) for the typical group; the difference in the number of reported NA responses for a participant was significant (*Mann-Whitney U*, *p* < 0.001) confirming the need to account for the NA response when comparing to neurotypical children to prevent bias.

### Applicable Responses

**FIGURE 1:**
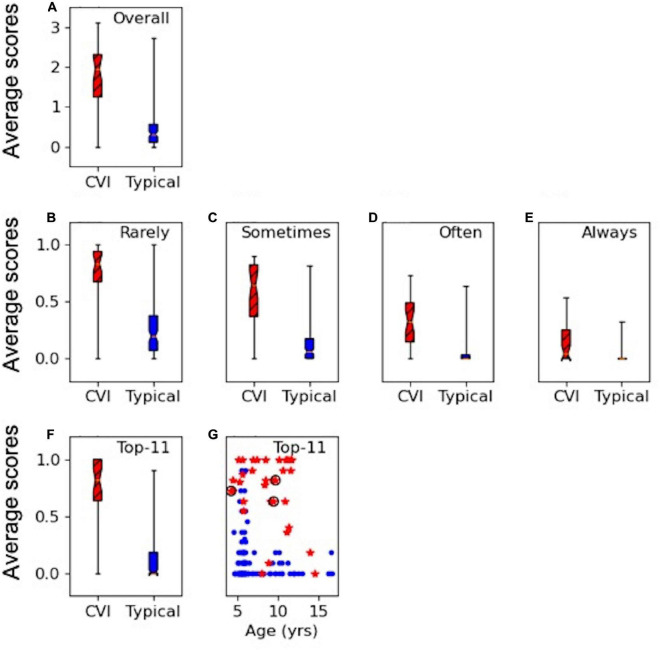
HVFQI-51 scores for CVI (in red and hatched) and typical (in blue) groups. Boxplots show 25th and 75th percentile of group data with the median shown as the narrowest part of the box; whiskers show data range. **(A)** Full Likert overall scores for HVFQI-51. **(B–E)** Scores for HVFQI-51 for “Rarely,” “Sometimes,” “Often,” and “Always” dichotomies (see Table 3). **(F)** Scores for the Top-11 subset of HVFQI-51. **(G)** Individual scores for the Top-11 subset of HVFQI-51 for typical children (blue dots) and children with CVI (red stars) as a function of age (in years); data for 3 children whose binocular visual acuity was slightly worse than 0.2 are marked by black rings.

Figures 1A–E show the results of the overall average score for the applicable responses on the HVFQI-51. The full 5-level Likert score result is shown in Figure 1A and scores for different cut-off threshold dichotomous scoring methods in Figures 1B–E. For all scoring methods, the CVI and the typical group were significantly different (Mann-Whitney U *p*-values < 0.001): the average scores were higher for children with CVI compared to typical children regardless of which scoring method was used.

The dichotomies based on cut-off thresholds at “Rarely,” “Often,” and “Sometimes” performed equally well to indicate HVFDs in the CVI group (see Supplementary Material for a detailed analysis). Therefore, we chose the top five most discriminant questions from each of these three dichotomy thresholds (in line with Dutton’s Five questions; Dutton et al., 2010b); i.e., a total of 15 questions. Four questions occurred in more than one dichotomy threshold. This yielded a total of 11 questions which we will refer to as the “Top-11” (Table 4). Figures 1F,G shows scores for the Top-11 for each group and also for individuals as a function of age indicating that Top-11 can potentially be used as a screening tool or a CVI-related impairment measure across the age range in children with CVI and good visual acuity, subject to further cross validation studies. Please note that for the questions in Top-11, we chose the cut-off for dichotomy according to which dichotomy level yielded the highest discriminability for that question (see Supplementary Material). For our sample of typical children and children with CVI, our Top-11 performs better than the 95 percentiles of randomly chosen sets of questions (with a similar procedure to make the set, or a similar set size); therefore, we suggest that the Top-11 set is a good potential for a screener. Nevertheless, we were limited by our sample size: our sample of typical children and children with CVI may not represent all the variation in the true population of children with CVI or typical children. We therefore caution the reader that we do not have sufficient statistical power to prove that the Top-11 set is the best set of questions for a screener tool; see Supplementary Material for more details. Further studies with independent samples are required to validate the Top-11.

**TABLE 4:** The Top-11 subset of the HVFQI-51.

Dichotomy cut-off threshold level	Q#	Question	Dutton’s Five (2010)	Dutton’s conceptual visual cognition domain
**Often**	19	Does your child have difficulty seeing something that is pointed out in the distance?	+	Complex scene (dorsal stream)
**Often**	29	Does your child find uneven ground difficult to walk over?		Visually guided movement (dorsal stream)
**Often**	39	Does your child bump into things when walking and having a conversation		Visual attention (dorsal stream)
**Often**	2	Does your child have difficulty walking downstairs	+	Visual field/attention to one side
**Often**	38	After being distracted does your child find it difficult to get back to what they were doing?		Visual attention (dorsal stream)
*Sometimes*	*2*	*also in Often*		As above
**Sometimes**	20	Does your child have difficulty finding a close friend or relative who is standing in a group		Complex scene (dorsal stream)
**Sometimes**	27	Does your child find copying words or drawings time-consuming and difficult	+	Complex scene (dorsal stream)
**Sometimes**	4	Does your child trip at the edges of pavements going down?		Visual field/attention to one side
*Sometimes*	*19*	*also in Often*		As above
*Rarely*	*2*	*also in Often and Sometimes*		As above
**Rarely**	34	Does your child find inside floor boundaries difficult to cross?		Complex scene (dorsal stream)
**Rarely**	14	Does your child have difficulty seeing scenery from a moving vehicle?		Perception of movement
*Rarely*	*20*	*also in Sometimes*		As above
**Rarely**	6	Does your child look down when crossing floor boundaries?		Visual field/attention to one side

*Q# indicates the question number in the HVFQI-51. They include the 5 questions that yielded the highest discriminability for the “Often,” “Sometimes,” and “Rarely” dichotomies; repeated questions are italicized and cross- referenced. Dutton (2010) suggested 5 questions (Dutton’s Five) for screening 3 of which are amongst the Top-11 are in the last column. Two items from Dutton (2010) are not included: 18—does your child have difficulty seeing things which are moving quickly, such as small animals; and 24—does your child have difficulty locating an item of clothing in a pile of clothes.*

#### Questions With Maximum Discriminability

**FIGURE 2:**
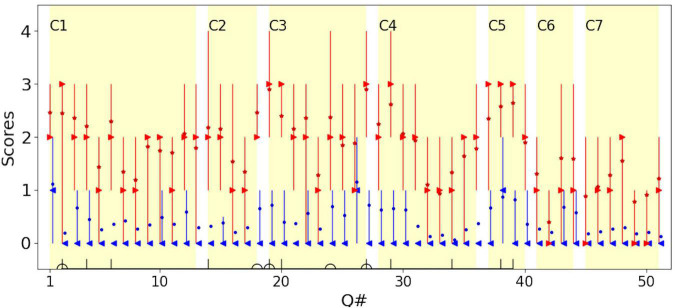
The distribution of scores for each question for children with CVI (red) and typical children (blue). For children with CVI, the median is shown in red right-pointing triangles; the mean is shown in red stars. For typical children, the median is show in blue left-pointing triangles; the mean is shown in blue dots. Error bars represent the 25th and 75th percentiles. To avoid overlapping of the plots, data for typical children is moved rightward by 10% of the unit. C1–C7 mark the 7 categories in Dutton et al. (2010). Questions in the Top-11 (vertical lines) and Dutton’s Five (half circles) are marked on the horizontal axis (see Table 4).

Figure 2 shows the scores for each question for children with CVI and for the typical group, distinguishing typical children (in blue) from children with CVI (in red) and displaying the variability of HVFDs in children with CVI and typical children. It can be seen that there are marked differences in both the median scores (observed frequency of behaviors) and the variability of scores in individual question items, between and within the CVI and the typical children groups.

For comparison, the seven conceptual categories, defined by Dutton et al. (2010b) as domains of visual cognition, that could be affected by CVI, are marked (C1:Visual field, C2: Perception of movement, C3: Visual search, C4: Guidance of movement, C5: Attention, C6: Crowded/complex scenes, C7: Recognition and navigation). Dutton et al. (2010) attributed C3, C4, and C5 to dorsal stream dysfunction and C7 to ventral stream dysfunction. In accordance with other studies, dorsal stream dysfunction was predominant in our group of children with CVI. For typical children, on average the score was less than 1 for both dorsal and ventral stream domains, i.e., for both they reported “Never” observed. For children with CVI, the average scores on questions attributed to the dorsal stream were close to 2 (i.e., a report of “Sometimes” observed) and for the ventral stream, were close to 1 (i.e., a report of “Rarely” observed). This confirms that behaviors suggesting dorsal stream dysfunction are more frequently reported than ventral stream dysfunction, at least in the group of children with CVI recruited to our study (Mann-Whitney *U*-test showed that the difference between the dorsal and ventral stream dysfunctions was significant in children with CVI; *p*-value < 0.001).

The Top-11 questions are marked by vertical lines on the horizontal axis in Figure 2. Note that the Top-11 questions are not necessarily the question items that give the highest median or mean scores (most frequently observed behaviors) in the group of children with CVI, but rather the most discriminant items from typical children for the corresponding dichotomy threshold. This allows for inclusion of even infrequently observed behaviors suggestive of HVFDs in the group of children with CVI, (such as question item 34, “Does your child find inside floor boundaries difficult to cross?”), which have discriminant value if these behaviors are usually never observed in typical children.

Figures 1F,G suggest that the scoring on the Top-11 (which is based on the criteria in Table 4) is clearly different between the typical children and children with CVI so that a threshold may be used to warrant further investigation. The decision on which threshold to use depends on false positive and true positive rates. Table 5 shows these indices for when the threshold is set variably at 4–8 questions endorsed out of the Top-11 (if all 11 questions are applicable).

**TABLE 5:** True and false positive rates for the Top-11 in our group of children with CVI and typical children, based on decision threshold.

# of endorsed questions in Top-11	8	7	6	5	4
Decision threshold: The score on Top-11 if all questions are applicable	0.72	0.64	0.55	0.45	0.36
True positive rate (sensitivity%)	66%	79%	82%	82%	88%
False positive rate (100-specificity%)	3%	5%	6%	7%	10%

### Internal Consistency of Dichotomous Scoring With Accounting for Not Applicable Responses

We calculated internal consistency taking into consideration NA responses for the three dichotomies scoring of Rarely, Sometimes and Often (see Supplementary Material for reasons why we chose these dichotomies). We used the Kuder–Richardson Formula 20 (KR-20) formula to assess reliability (Equation 1; Kuder and Richardson, 1937) for the dichotomous scoring methods with an adjustment for the NA responses to prevent underestimation of internal consistency given that the variance of total scores ($\sigma_{t}^{2}$) would be underestimated.


(1) $$KR\_20=\frac{K}{K-1}\,\left[1-\frac{\sum_{i=1}^{K}p_{i}\,q_{i}}{\sigma_{t}^{2}}\right]$$

Where K is the number of questions. To account for the under-estimation related to NA answers, we used an “adjusted total variance” where the “total score” for a given participant is scaled up by a factor of $\frac{\text{To}\mathrm{tal}\#\mathrm{of}\mathrm{questions}\left(\mathrm{incl}.\mathrm{NA}\right)}{\#\,o\,f\,a\,p\,p\,l\,i\,c\,a\,b\,l\,e\,q\,u\,e\,s\,t\,i\,o\,n\,s}$ (Arifin and Malaysia, 2018). Therefore, in Equation 1, ($\sigma_{t,a\,d\,j\,u\,s\,t\,e\,d}^{2}$) was used instead of ($\sigma_{t}^{2}$). In addition, we calculated *p*_*i*_ as the proportion of endorsed and *q*_*i*_ as the proportion of not endorsed responses for the applicable responses. The adjusted KR_20 for the dichotomy at “Rarely,” “Sometimes,” and “Often” analysis was 0.978, 0.978, and 0.968, respectively, indicating high internal consistency and reliable scoring for all three dichotomies.

## Discussion

Our prospective study confirms that the HVFQI-51 clearly distinguishes the range of visually guided behaviors in children with an established clinical diagnosis of CVI *and* good visual acuity from neurotypical children. Our study characterizes the spectrum of visual perceptual difficulties in this unique cohort of children as largely “dorsal stream” deficits with the most discriminatory questions distinguishing the normal range of visually guided behaviors observed in typical children outlined in Table 4. We thus identify a subset of 11 questions, the Top-11, that appear to be most discriminative for HVFDs in children with CVI and good visual acuity compared to typical children. In addition, to ensure an accurate representation of HVFDs within the overall disability, we account for the NA responses within the analysis and suggest that they be included as a response option and analyzed in further studies.

High internal consistency for questions indicates that they are correlated and the reliability is reflected by the excellent overall value of a minimum of 0.968 or higher as we confirm with our analysis of division of responses along the dichotomy scoring method. High internal consistency has been reported previously in studies using similar and different inventories in children with CVI (Macintyre-Beon et al., 2012; Pueyo et al., 2014; Salavati et al., 2015; Philip et al., 2016; Gorrie et al., 2019). Direct comparisons with other works using a questionnaire to study children with an independent diagnosis of CVI are difficult because of difference in study designs and different question inventories. A similar study, by Macintyre-Beon et al. (2012) used the 51-question inventory (CVI-I) on a similar number of children with CVI (*n* = 36), established an independent clinical diagnosis of CVI and included a control group (*n* = 156). There are differences from their study, particularly in their unstated extent of visual acuity loss, their dichotomy of the Likert scale into normal and impaired based on a single cut-off (“Often”) value, use of Cronbach’s alpha for reliability measured for subgroups of questions (grouped on presumed neurobiologically feasible conceptual domains of visual cognition; i.e., the 7 categories presented; Figure 2), rather than individual question items, and no reported analysis of NA responses. Furthermore, Macintyre-Beon et al. (2012) did not present an analysis of the variability of responses between and within typical children and children with CVI in the groups of children in their study, or an analysis of the discriminant values of the individual questionnaire items. Notwithstanding these differences, the HVFQI-51 in our study reports similar high internal consistency indicating that the HVFQI-51 is clearly reliable at detecting HVFDs even in children with good visual acuity.

We suggest the Top-11 is a potentially discriminative tool for measuring HVFDs in children with CVI, i.e., a measure of CVI-related impairment. The Top-11 discriminating question items from our results (Figures 1, 2) covers a range of behaviors suggesting HVFDs as generally reported for children with CVI (Dutton et al., 1996; Bax, 2010; Jackel et al., 2010; Sakki et al., 2018; Gorrie et al., 2019; Jackel, 2019; Lueck et al., 2019). The 11 most discriminating questions elicited HVFDs in awareness of lower visual field, distance viewing, finding objects in environmental clutter, multiple task management (central attention) and motion perception despite good visual acuity in these children. A previous study of children with CVI with a wide range of acuity loss (Dutton et al., 1996) identified simultaneous perception, perception of movement, orientation, recognition and depth perception as being the most frequent HVFDs. These are similar to our cohort with good visual acuity indicating independence of HVFDs from visual acuity measures and reliability of the HVFQI (see Figure 2) and, the potential reliability of our derived Top-11 (see Table 4) in eliciting HVFDs.

Studying hospital records, using a Flemish question inventory based on work by Ortibus et al. (2011) and Ben Itzhak et al. (2019) suggests object and face processing impairment (ventral stream deficit) visual (dis)interest, clutter, distance viewing, and moving in space were factors that distinguished children with a CVI from a non-CVI diagnosis. Gorrie et al. (2019) used the CVI Questionnaire (Ortibus et al., 2011); a survey with 46 items with Yes/No options for parents. 539 children (age 5–18 years) were included; the diagnosis of CVI was based on parental report. 104 children were reported to have CVI, although data on visual acuity was mostly not provided. Gorrie et al. (2019) used an exploratory factor analysis. The following five factors were significant, with given items contributing to more than one factor: (i) F1, complex neurological problems, (ii) F2, dorsal and ventral stream functions, (iii) F3, visual attention, (iv) F4, influence of a familiar environment on vision, and (v) F5, processing in multi-task activities. Children in our study, on the other hand, had a confirmed CVI diagnosis and had good visual acuity. The sample size in our study was not sufficient for us to perform a factor analysis (51 items, 5-level Likert and NA options). Nevertheless, the Top-11 could cover factor 2, 3, and 5. Further studies are needed with a more diverse and larger cohort of children with CVI to derive underlying neurobiologically-feasible factors in the HVFQI-51 using a data-driven approach; and whether these factors correspond with the theory-driven subgroups of questions in Macintyre-Beon et al. (2012) and the data-driven factors of Gorrie et al. (2019).

The results of our study provide novel information on the range of frequency of observation for each putative HVFD questionnaire item which we note do not necessarily indicate measuring a visual pathway problem, since motor and cognitive problems independent of visual pathways can cause the same behavior. The HVFQI-51 items span a wide spectrum of potential visually guided behaviors, and our study identifies the range of such behaviors in typical children as well as in a sample of children with CVI. It should be noted that it is the conjunction of items and responses rather than the individual item response in isolation that indicates that the child’s behaviors are the result of a visual pathway disruption. The information on the range of behaviors suggesting HVFDs in controls (typical children) allows us to compare to the range of frequency of that item in children who are known to be abnormal with CVI, usually combined with other neurodevelopmental comorbidities since a brain network disruption rarely affects visual pathways only. This provides information on how discriminant each item is, and thus how much weight to place on a response in that item when considering CVI-related impairment. It is an essential part of clinical assessment and history-taking in patient populations to know the range of behaviors that are normal or appear to be abnormal in normal children and hence, are not actually abnormal behavior, but simply within the range of normal behavior in the childhood non-clinical population. The discriminant value of each item in the HVFQI-51 is important information not just for using that item or a subset of items (i.e., Top-11) as a diagnostic tool, but also for allowing a quantification of CVI-related impairment (see e.g., Tsirka et al., 2020, who used the Insight inventory for measuring CVI-related impairment before and after intervention strategies). Some question items should have more weight than others in measuring CVI-related impairment, i.e., the items with most discriminant value from normal range of behavior, rather than simply summing the responses across all items equally irrespective of their discriminant value as done by Sakki et al. (2020) who used the 51-question CVI-I. We propose in effect the Top-11 has these favorable properties: a range of visually guided behaviors across conceptual domains of visual cognition, but importantly discriminate from behaviors seen in typical children. The Top-11 thus has potential as not just a “screener” implying a short diagnostic tool of CVI for individual child-level clinical practice, or as used in large scale epidemiological studies where many hundreds of participants need to be screened, as Dutton’s Top 5 has been used by Gorrie et al. (2019), Williams et al. (2021), but also as a *scale* to measure severity of CVI-related impairment in children diagnosed with CVI (in the way the Insight inventory was used by Tsirka et al., 2020). We would re-emphasize that we consider the Top-11 as a potential tool subject to further studies and do not recommend its widespread clinical use yet.

We did not find a significant number of putative ventral stream deficits in our CVI group, though other impairments suggestive of dorsal stream dysfunction are in common with our Top-11. Categorical descriptions are difficult as most HVFDs engage multiple HVFs across both dorsal and ventral streams, other sensory processing and integration with motor commands (Merabet et al., 2017; Milner, 2017). However, based on our study and other reports there are HVFDs common in CVI encapsulated within the Top-11. Further studies are needed to establish the reliability of the Top-11 in a secondary independent sample. As an aid to those studies a threshold which activates taking a detailed HVFQI-51 would be helpful. This threshold will depend on acceptable true and false positive rates (see Table 5). A threshold that gives at least 70–80% true positive rate outcome is likely to ensure that at-risk children are not missed (see Ortibus et al., 2011).

van Genderen et al. (2012) chose 12 questions, from a more extensive questionnaire (Houliston et al., 1999), based on problems reported more often in children with CVI compared to children with a suspected diagnosis of CVI. They found the 12-item question inventory with only Yes/No/Sometimes responses not sensitive to diagnosing CVI in children with good visual acuity suspected of CVI. Our Top-11 includes five of their 12 questions (Q# 2, 4, 14, 20, and 29) but also an additional six questions. The differences could lie within the wider set of Likert response for each question, additional accounting for the NA option and a different methodology for endorsing responses in our study to make the Top-11.

We suggest that HVFQI-51, its derivative Top-11 and possibly other similar question inventories, have utility in characterizing the range of functional impairments related to higher visual function affecting the individual child and identifying strategies for treatment. Although these question inventories can be used to support a clinical diagnosis of CVI, these tools are not suitable for diagnosing CVI on their own. This is because these functional impairments, presumptively due to HVFDs, may be present in other conditions, such as developmental co-ordination disorder (Chokron and Dutton, 2016) or autism spectrum disorder, which may be co-morbid with CVI (Ortibus et al., 2019; Chokron et al., 2020; Molinaro et al., 2020). In addition, visually guided behavior may be abnormal because of a dysfunction outside the visual pathway such as a motor pathway (Ghasia et al., 2008; Salavati et al., 2014). For example, tripping on a curb could be due to depth perception problems or a problem with ankle dorsiflexion. This is a particular challenge in children with cerebral palsy and periventricular leukomalacia since they are likely to have both CVI and cerebral motor pathway impairment, the latter defining cerebral palsy. Tools to understand the association and interdependence between visual and motor dysfunction are being developed and used for identifying these aspects (Salavati et al., 2017; Sakki et al., 2021). The HVFQI-51, Top-11 and other question inventories may be used in children with suspected CVI and could form an important complementary role to other clinical evaluation for the assessment of CVI within the context of risk factors and coexisting conditions.

In our study, the diagnosis of CVI was established independent of the HVFQI-51. Though the nomenclature and diagnosis of CVI has a contentious history (Jan et al., 2001; Deonna and Roulet, 2004; Jacobson et al., 2004; Matsuba and Jan, 2006), the diagnosis of CVI in our study essentially remains a clinical one based on a multidisciplinary approach between ophthalmology, neurology and where necessary brain neuroimaging (Dutton and Jacobson, 2001; Signorini et al., 2005; Fazzi et al., 2007; Ospina, 2009; Roman et al., 2010; Lueck et al., 2019; Ortibus et al., 2019; Sakki et al., 2020). Neuroimaging was done in most of our CVI cohort (31/33) and in 7 children the MRI was judged to be normal. Neuroimaging or indeed an abnormal MRI scan is currently not considered essential for diagnosis of CVI (Salavati et al., 2015). Absence of abnormalities on MRI brain scan does not exclude CVI as abnormalities may not be seen due to current limitations of image resolution (Ortibus et al., 2009, 2019) and even a normal MRI brain postdating abnormal neonatal ultrasonography at birth (van Genderen et al., 2012) does not exclude CVI. Some studies have relied on inventories to diagnose CVI and recommend their use for diagnosis (Ortibus et al., 2011; Macintyre-Beon et al., 2013; Philip et al., 2016; Gorrie et al., 2019) or, only on parental reports of diagnosis without corroboratory evidence (Gorrie et al., 2019). The Insight inventory and later CVI-I originally published by Dutton et al. (2010b) though originally developed for children with CVI was never meant to diagnose CVI, only to document cerebral visual dysfunction (Bax, 2010; Macintyre-Beon et al., 2012) which in itself may be a component of other brain-based conditions associated with visual behaviors similar to those with CVI. The ideal diagnostic criteria which encompass this protean condition of CVI have not yet been established (Sakki et al., 2018). Therefore, we have used the term HVFQI-51 (rather than CVI-I) to emphasize that the role of HVFQI-51 (rather than the CVI-I) is to document HFVDs and not establish a diagnosis of CVI as the only measure (van Genderen et al., 2012).

Questionnaires like the HVFQI-51 often comprise questions that may be critical for the purpose but do not necessarily apply to every respondent (Frary, 2003) due to physical or cognitive limitations and often include a “Not Applicable” (NA) response option. The NA option is useful and extends the applicability to a wider group of children with CVI as they often have a wide spectrum of physical and perceptual deficits. For example, for a child in a wheelchair, questions such as difficulties in “tripping over pavement” and “coming down stairs” may not be applicable. The NA option has been part of previous versions of the inventory (CVI-I and Insight) and was retained for the HVFQI-51. However, in previous studies, NA responses have not been reported (Gorrie et al., 2019); or items with a high rate of NA responses were excluded (Tsirka et al., 2020) not accounted for Gorrie et al. (2019) or not mentioned (Macintyre-Beon et al., 2012; Philip et al., 2016). If NA responses are not accounted for, i.e., counted as not endorsed, the question inventory score will be artificially low, reducing the chance of further investigations for HVFDs. The other extreme is to count all of NA as endorsed which will artificially increase the score for children who may not have HVFDs. A variety of ways to account for the NA response in question inventories have been suggested in the literature (Fayers et al., 1998; Holman et al., 2004). Here, we implement one of the methods, modified specifically for the HVFQI-51 to analyze the NA responses within the total number of questions for each subject allowing us to use all the data (Sakki et al., 2020). We believe this is essential to provide a holistic picture of the child with CVI and importantly to remove any bias when comparing the two groups in our study as NA responses were significantly higher, as expected, in the CVI group compared to the typically developing group.

Our study does have limitations. Dorsal and not ventral stream dysfunction is characterized by our study population. Our clinical cohort of children with CVI and good acuity largely comprised etiologies known to lead to patterns of dysfunction processed through the commonly affected dorsal stream (see Dutton, 2009 for a review) and dorsal stream has been reported to be more vulnerable to cerebral insults during early visual development (Atkinson and Braddick, 2005). Other etiological mechanisms of brain injury such as temporal lobe lesions (encephalitis, tumor, hemorrhage, rare calcification syndromes) may yield a different pattern of ventral stream-related HVFDs.

We provide detail of comorbidities from an independent neurological examination and neuroimaging results. We acknowledge that the questions can also pick up motor (efferent) pathway abnormality rather than visual (afferent) pathway abnormality since many children have comorbid motor problems—either gross motor problems such as cerebral palsy, or milder problems in balance. There are some questions which do not appear to have any motor component, such as finding faces in a crowd whereas others are likely to load significantly on motor problems even if there are depth perception problems or visual field problems such as bumping into objects or tripping on the curb. One study (van Genderen et al., 2012) did find an overlap between HVFDs in CVI and good visual acuity and comorbid conditions with an abbreviated 12 question, 3-choice inventory. Our results for a similar population are different possibly because of design differences and we have employed a 51 question inventory with 5-response options.

Our population of children with comorbid conditions is typical of a clinical population of children with CVI. It is similar to other published studies using either question or assessment inventories documenting HVFDs and visual perceptual problems where comorbidities have been documented (Fazzi et al., 2007; Roman et al., 2010; Chong and Dai, 2014; Gorrie et al., 2019; Ortibus et al., 2019; Ben Itzhak et al., 2020; Chokron et al., 2020; Tsirka et al., 2020). Sakki et al. (2018) and also acknowledge the difficulties and controversy in diagnosing HVFDS in CVI in presence of co-morbidities and further outline a spectrum of HVFDs that relate to CVI similar to those documented in our study. Moreover, HVFQI-51 is designed to ask questions related to visual perceptual problems and not cognitive problems which may be a predominant feature in some of the comorbidities such as autism. Finally, the relatively small cohort of children in our study is offset by the prospective design, an independent established diagnosis of CVI, a population of CVI with near-normal visual acuity and comparison with a control group.

In summary, the HVFQI-51 is a potentially useful assessment tool for characterizing HVFDs in children with CVI when compared to normal children and applicable to similar cohorts with behaviors suggesting HVFDs. The set of Top-11 questions derived from HVFQI-51 has the potential to serve as a screening tool and as a CVI-related impairment measure, feasible to use in routine clinical practice or for larger scale studies. High scores on the Top-11 should instigate more detailed characterization of HVFDs with the longer and more detailed HVFQI-51 which covers a wider spectrum of visually guided behaviors. The HVFQI-51 (and, potentially the Top-11 once validated by further studies with independent samples) can also be applied in clinical practice for evaluating children with a history of brain damage or disruption of brain development where there are concerns at home or school about abnormal visual function but visual acuity is good. Our results confirm that poor visual functioning in normal environments and at school in the presence of good visual acuity or a normal ocular examination should engender a high index of suspicion of the possibility of HVFDs in CVI (Macintyre-Beon et al., 2013; Williams et al., 2021). Williams et al. (2021) using the CVI-I of Macintyre-Beon et al. (2012) reported that in mainstream schools on average one child in a class of 30 children has one or more CVI-related vision problems, with most (79%) being already identified as at-risk, thus delineating a group that may benefit form screening for CVI.

Future work will focus on validation of HVFQI-51 and the Top-11 with a larger set of patients; with a wider set of disabilities and within homogeneous radiological or clinical subgroups (e.g., occipital lobe injuries visible on MRI or children with periventricular leukomalacia); determining usefulness as a screening tool and as a CVI-related impairment measure, and studying the long-term natural history of HVFDs in CVI with and without targeted intervention. Additional separate work is needed to assess the place of question inventories within group of children with disabilities without evident clinically diagnosed CVI and with other tests of visual perception such as visual evoked potentials (Weinstein et al., 2012) and standard neuropsychological tests (Tsirka et al., 2020).

## Data Availability Statement

The raw data supporting the conclusions of this article will be made available by the authors, without undue reservation.

## Ethics Statement

The studies involving human participants were reviewed and approved by the NHS Research Heath Authority IRAS ID:193481; REC Reference:16/EE/0062. The patients/participants provided their written informed consent to participate in this study.

## Author Contributions

AC conceptualized the study and performed the eye examinations. AC and SF collected the data. RK wrote the neurological sections and did the neurological examinations and interpretation of the brain imaging. SG did the data analysis and produced the figures. AC was the lead in writing the manuscript with SG who wrote most of the data analysis section. All authors contributing to the editing and final review of the manuscript.

## Conflict of Interest

The authors declare that the research was conducted in the absence of any commercial or financial relationships that could be construed as a potential conflict of interest.

## Publisher’s Note

All claims expressed in this article are solely those of the authors and do not necessarily represent those of their affiliated organizations, or those of the publisher, the editors and the reviewers. Any product that may be evaluated in this article, or claim that may be made by its manufacturer, is not guaranteed or endorsed by the publisher.

## Footnotes

1
http://www.python.org
