# Supplementary material for: Higher Visual Function Deficits in Children With Cerebral Visual Impairment and Good Visual Acuity
Source: Front Hum Neurosci. 2021 Nov 16;15:711873. doi: 10.3389/fnhum.2021.711873 (PMC8636735; doi:10.3389/fnhum.2021.711873)
Supplement: Supplementary file 1 [file Data_Sheet_1.PDF]

About your child

Participant code:_________________________

How many weeks into your pregnancy were you when you gave birth?

____

Were there any problems at the time of birth? If so, please describe:

____

____

Has your child had any conditions affecting the eyes or brain? If so, please describe:

____

____

Do you have any concerns about your child's vision? If so what are they?

____

____

Instructions to parents

We want to find out what children at different ages can and cannot see. We want to know about how your child behaves **now** but not when younger.

These questions are designed for a range of ages, so some questions may seem odd. Your child may have difficulty with some behaviours listed below but not others – this is normal.

Also you may notice that some of the behaviours described occur occasionally when your child is tired, this is common.

For each of the items listed, please could you tick the box which best fits with your child’s **present** behaviour.

never/ rarely/ sometimes/ often/ always/ not applicable (NA)

Does your child...

1. trip over toys and obstacles on the floor?
2. have difficulty walking down stairs?
3. trip at the edges of pavements going up?
4. trip at the edges of pavements going down?
5. appear to ‘get stuck’ at the top of a slide/ hill?
6. look down when crossing floor boundaries e.g. where lino meets carpet?
7. leave food on the near or far side of their plate?

If so, on which side? (near/far)

8. leave food on the right or left side of their plate?

If so, on which side? (right/left)

9. have difficulty finding the beginning of a line when reading?
10. have difficulty finding the next word when reading?
11. walk out in front of traffic?

If so, which side? (right/left/both)

12. bump into doorframes or partly open doors?

If so, which side?(right/left/both)

13. miss pictures or words on one side of a page?

If so, which side? ?(right/left/both)

14. have difficulty seeing scenery from a moving vehicle?
15. have difficulty seeing things which are moving quickly, such as small animals?
16. avoid watching fast moving TV?
17. choose to watch slow moving TV?
18. have difficulty catching a ball?
19. have difficulty seeing something which is pointed out in the distance?
20. have difficulty finding a close friend or relative who is standing in a group?
21. have difficulty finding an item in a supermarket e.g. finding the breakfast cereal they want?
22. get lost in places where there is a lot to see, e.g., a crowded shop?
23. get lost in places which are well known to them?
24. have difficulty locating an item of clothing in a pile of clothes?
25. have difficulty selecting a chosen toy in a toy box?
26. sit closer to the television than about 30cm if you let him/her.
27. find copying words or drawings time consuming and difficult?
28. when walking hold onto your clothes, tugging down?
29. find uneven ground difficult to walk over?
30. bump into low furniture such as a coffee table?
31. bump into low furniture if it is moved?

32. get angry if furniture is moved?

33. explore floor boundaries (e.g. lino/carpet) with their foot before crossing the boundary?

34. find inside floor boundaries difficult to cross?

34.a. If so…boundaries that are new to them?

34.b. If so...boundaries that are well known to them?

35. reach incorrectly for objects, that is, do they reach beyond or around the object?

36. when picking up an object grasp incorrectly, that is do they miss or knock the object over?

37. find it difficult to keep to task for more than 5 minutes?

38. after being distracted find it difficult to get back to what they were doing?

39. bump into things when walking and having a conversation?

40. miss objects which are obvious to you because they are different from their background and seem to 'pop out' e.g. A bright ball in the grass?

41. Do rooms with a lot of clutter cause difficult behaviour?

42. Do quiet places/open countryside cause better behaviour?

43. Is behaviour in a busy supermarket or shopping centre difficult?

44. react angrily when other restless children cause distraction?

45. have difficulty recognising close relatives in real life?

46. have difficulty recognising close relatives from photographs?

47. mistakenly identify strangers as people known to them?

48. have difficulty understanding the meaning of facial expressions?

49. have difficulty naming common colours?

50. have difficulty naming basic shapes such as squares, triangles and circles?

51. have difficulty recognising familiar objects such as the family car?

Thank you very much for your time!
