# Supplementary material for: Higher Visual Function Deficits in Children With Cerebral Visual Impairment and Good Visual Acuity
Source: Front Hum Neurosci. 2021 Nov 16;15:711873. doi: 10.3389/fnhum.2021.711873 (PMC8636735; doi:10.3389/fnhum.2021.711873)
Supplement: Supplementary file 2 [file Data_Sheet_2.docx]

# Our Top-11 set

To investigate the performance of the HVFQI-51 and, for the analysis of the most discriminatory questions, ROCs were derived for each dichotomy scoring method (Table 3) based on (i) the score on the HVFQI-51 and (ii) whether the child had a CVI diagnosis. The best dichotomy level was chosen based on the ROC curves and the top 5 most discriminant questions from the best dichotomy were chosen for the likelihood of a potential screener (in line with Dutton’s Five questions).

Figure S1 shows the ROC curves for different dichotomy methods. Area under the curve (AUC) for the overall full Likert scores and for the 4 dichotomy methods of ‘Rarely’, ‘Sometimes’, ‘Often’, and ‘Always’ were 0.872, 0.861, 0.876, 0.886 and 0.815, respectively. These scores are considered ‘excellent’ indicating good discrimination ability of the HVFQI-51 (1). However, for the dichotomy methods, the ROC curve for the ‘Always’ dichotomy was lower than the other curves while the three curves for the remaining dichotomies were similar. Traditionally, the ROC with the highest AUC is chosen. Given how similar the ROC curves and AUC values are for three of the four dichotomies and, given the sample size in our study, we were not comfortable to just choose the highest value dichotomy level without further investigation. To determine whether they were significantly different, we ran a bootstrap analysis (1000 runs) for the three ROCs which showed considerable overlap. The estimated bootstrap standard deviations for AUC for ROCs based on Rarely, Sometimes, and Often divisions were 0.04, 0.04, and 0.03, respectively. Based on these standard deviations, we concluded that the three dichotomies yielded similar AUCs (see also 2). Therefore, for the rest of the paper, our analysis is based on these three dichotomies.

We chose the top 5 questions with highest AUC from each level of the three dichotomies: first from ‘Often’, then ‘Sometimes’, and finally ‘Rarely’ dichotomy. The results revealed a set of 11 questions (Top-11 HVFQI); see Table 4). The ROC for Top-11 HVFQI is shown in Figure S1 with an AUC of 0.922 and with bootstrapping (1000 run) standard deviation of 0.04.


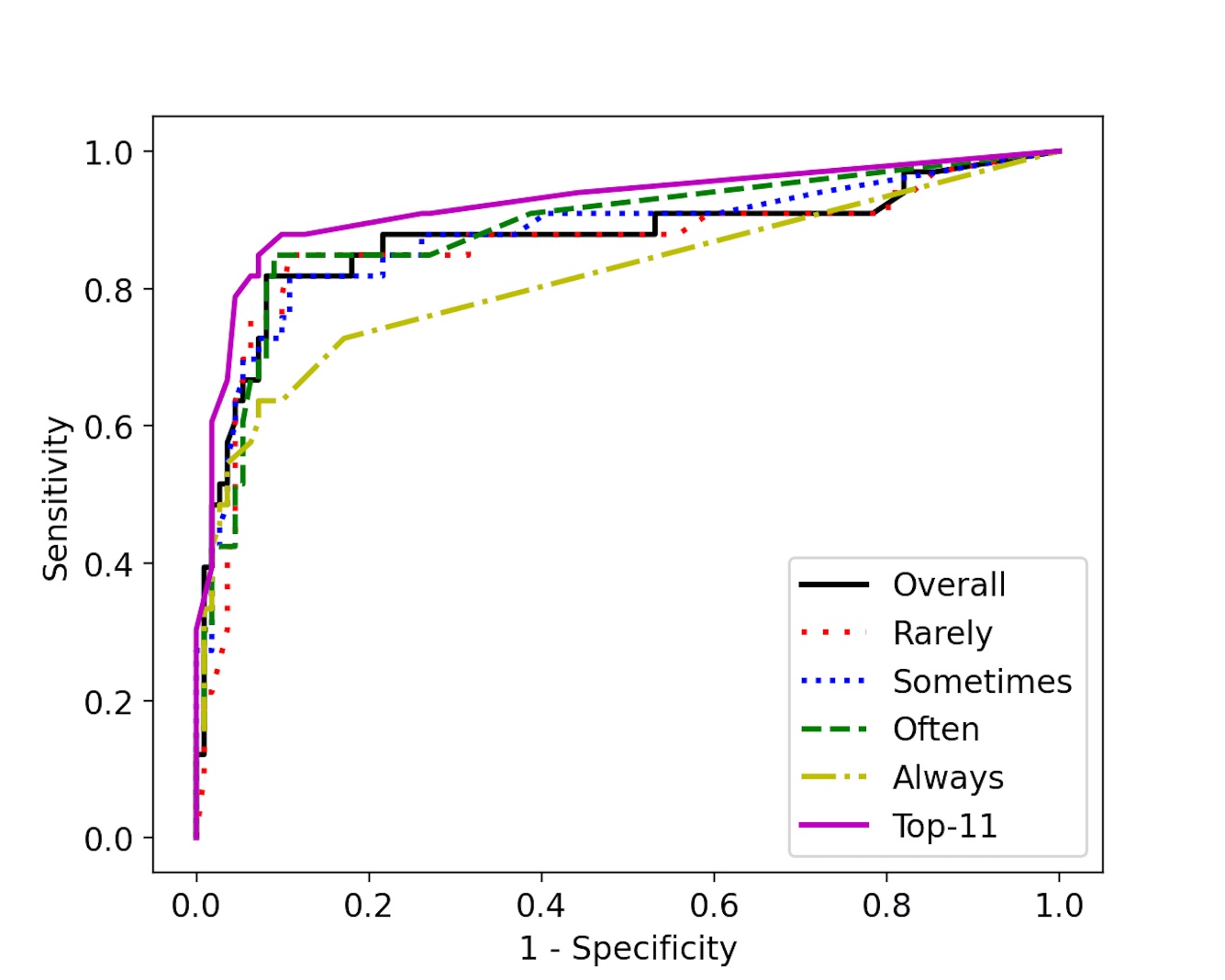


Figure S1. ROC curves for binary classification (CVI vs Typical) based on scores on HVFQI-51. Results are plotted for the overall full Likert scores, different dichotomy scores, and for the Top-11 subset of the HVFQI-51.

# How is the performance of our Top-11 compared to a set of randomly-selected questions in our sample?

Furthermore, to ensure that we had chosen the questions with the highest ‘discriminability’, we investigated the performance of the 11 questions against a set of randomly-selected questions.

In brief, we had chosen the 5 questions that yielded the highest discriminability from each of the 3 dichotomies (‘Rarely’, ‘Sometimes’ and ‘Often’); these questions made the Top-11 (note: 4 questions were in more than one dichotomy). The Top-11 gave an AUC of 0.92.

To assess the performance of these eleven questions in comparison to other questions, we employed two strategies using iterative permutation analysis. In the first, we utilized the same method as the Top-11 but chose random questions. In the second we kept the set size at 11 questions and chose random questions.

## First strategy: Performance of the Top-11 when five questions are chosen ‘by random’ from each of the 3 dichotomies.

From each of the three dichotomies, we chose 5 questions ‘by random’ and without substitution. Then, we put together these questions to make a Test set. In our Top-11, there were 4 questions that were in more than one dichotomy; resulting in 11 final questions. Similarly, it is possible that there are questions in each Test set that belong to more than 1 dichotomy. Therefore, the size of Test sets could vary between 5 (an unlikely but possible scenario in which the same 5 questions are chosen by chance from each dichotomy) to 15 (a scenario in which there are no overlapping questions). In our permutation, the 5, 50 and 95 percentiles for ‘number of questions in a Test set’ were 12, 14 and 15 questions, respectively.

There are 11 questions in Top-11, which is less than the 5% of the observed Test-set size in our permutation analysis. This suggests that the questions in our Top-11 were informative rather than random questions and 4 of our questions had very high discriminability in more than one dichotomy. We calculated the AUC for the Test sets using the same procedure that we used for Top-11. We repeated this 10000 times to find the distribution of AUC for the Test sets. In this permutation, the 5, 50 and 95 percentiles for AUC for Test sets were 0.83, 0.86 and 0.89, respectively.

## Second strategy: Performance of the Top-11 when the Test set size is kept at 11 randomly selected questions

Dichotomy criteria similar to Top-11

In our Top-11 set, 5 questions were from the ‘Often’ dichotomy, 3 from the ‘Sometimes’ dichotomy, and 3 from the ‘Rarely’ dichotomy. In this strategy we followed a similar pattern. In each Test set of 11 questions we randomly assigned criteria of ‘Often’ to 5, ‘Sometimes’ to 3, and ‘Rarely’ to 3 questions. Then we calculated the AUC. We ran the permutation 10000 times. The 5, 50 and 95 percentiles for AUC for Test sets were 0.82, 0.86 and 0.89, respectively.

In the following, we find the distribution of AUCs for different scenarios for the scoring criteria.

‘Rarely’ dichotomy

In this analysis, we used the Rarely dichotomy criteria for all questions in the Test set. Then we calculated the AUC. We ran the permutation 10000 times. The 5, 50 and 95 percentiles for AUC for Test sets were 0.82, 0.85 and 0.87, respectively.

‘Sometimes’ dichotomy

In this analysis, we used the Sometimes dichotomy criteria for all questions in the Test set. Then we calculated the AUC. We ran the permutation 10000 times. The 5, 50 and 95 percentiles for AUC for Test sets were 0.81, 0.85 and 0.87, respectively.

‘Often’ dichotomy

In this analysis, we used the Sometimes dichotomy criteria for all questions in the Test set. Then we calculated the AUC. We ran the permutation 10000 times. The 5, 50 and 95 percentiles for AUC for Test sets were 0.83, 0.86 and 0.89, respectively.

Full Likert score

In this analysis, we used the full Likert score for all questions in the Test set. Note that we no longer used the dichotomy scoring. Then we calculated the AUC. We ran the permutation 10000 times. The 5, 50 and 95 percentiles for AUC for Test sets were 0.84, 0.86 and 0.89, respectively.

The Top-11 set of questions with a high AUC of 0.92 performs better than randomly selected sets in our sample of children with CVI and typical children, confirming it being a good potential candidate for screening for HVFDs and documenting CVI-related HVFDs.

Nevertheless, we are limited by our sample size. How do the results transfer to the true population of children with CVI? The true distributions of AUC for Test sets will not be the same as the distributions we found based on the sample of children with CVI and typical children in our study; there will be differences. The bootstrapping analysis (permutation with substitution over the CVI and Typical group; keeping the set of questions constant) showed that the estimated SD for AUC for Top-11 was 0.04. We do not claim that the Top-11 is the best set of questions for the screener, and we do not have the statistical power to prove it. Rather, we suggest that based on our sample and analysis, the Top-11 is a good potential candidate for a screener.

**References**

1. Hosmer DW, Lemeshow S. Applied Logistic Regression. 2 ed. New York, NY: John Wiley and Sons; 2000.

2. Mandrekar JN. Receiver operating characteristic curve in diagnostic test assessment. J Thorac Oncol. 2010;5(9):1315-6.
